# Assessing the Causal Relationship of Maternal Height on Birth Size and Gestational Age at Birth: A Mendelian Randomization Analysis

**DOI:** 10.1371/journal.pmed.1001865

**Published:** 2015-08-18

**Authors:** Ge Zhang, Jonas Bacelis, Candice Lengyel, Kari Teramo, Mikko Hallman, Øyvind Helgeland, Stefan Johansson, Ronny Myhre, Verena Sengpiel, Pål Rasmus Njølstad, Bo Jacobsson, Louis Muglia

**Affiliations:** 1 Human Genetics Division, Cincinnati Children’s Hospital Medical Center, Cincinnati, Ohio, United States of America; 2 Center for Prevention of Preterm Birth, Perinatal Institute, Cincinnati Children’s Hospital Medical Center and March of Dimes Prematurity Research Center Ohio Collaborative, Cincinnati, Ohio, United States of America; 3 Department of Obstetrics and Gynecology, Sahlgrenska University Hospital, Gothenburg, Sweden; 4 Obstetrics and Gynecology, University of Helsinki and Helsinki University Hospital, Helsinki, Finland; 5 PEDEGO Research Center, University of Oulu and Department of Children and Adolescents, Oulu University Hospital, Oulu, Finland; 6 KG Jebsen Center for Diabetes Research, Department of Clinical Science, University of Bergen, Bergen, Norway; 7 Center for Medical Genetics and Molecular Medicine, Haukeland University Hospital, Bergen, Norway; 8 Department of Genes and Environment, Division of Epidemiology, Norwegian Institute of Public Health, Oslo, Norway; 9 Department of Pediatrics, Haukeland University Hospital, Bergen, Norway; 10 Department of Obstetrics and Gynecology, Institute of Clinical Sciences, Sahlgrenska Academy, Gothenburg University, Gothenburg, Sweden; University of Manchester, UNITED KINGDOM

## Abstract

**Background:**

Observational epidemiological studies indicate that maternal height is associated with gestational age at birth and fetal growth measures (i.e., shorter mothers deliver infants at earlier gestational ages with lower birth weight and birth length). Different mechanisms have been postulated to explain these associations. This study aimed to investigate the casual relationships behind the strong association of maternal height with fetal growth measures (i.e., birth length and birth weight) and gestational age by a Mendelian randomization approach.

**Methods and Findings:**

We conducted a Mendelian randomization analysis using phenotype and genome-wide single nucleotide polymorphism (SNP) data of 3,485 mother/infant pairs from birth cohorts collected from three Nordic countries (Finland, Denmark, and Norway). We constructed a genetic score based on 697 SNPs known to be associated with adult height to index maternal height. To avoid confounding due to genetic sharing between mother and infant, we inferred parental transmission of the height-associated SNPs and utilized the haplotype genetic score derived from nontransmitted alleles as a valid genetic instrument for maternal height. In observational analysis, maternal height was significantly associated with birth length (*p* = 6.31 × 10^−9^), birth weight (*p* = 2.19 × 10^−15^), and gestational age (*p* = 1.51 × 10^−7^). Our parental-specific haplotype score association analysis revealed that birth length and birth weight were significantly associated with the maternal transmitted haplotype score as well as the paternal transmitted haplotype score. Their association with the maternal nontransmitted haplotype score was far less significant, indicating a major fetal genetic influence on these fetal growth measures. In contrast, gestational age was significantly associated with the nontransmitted haplotype score (*p* = 0.0424) and demonstrated a significant (*p* = 0.0234) causal effect of every 1 cm increase in maternal height resulting in ~0.4 more gestational d. Limitations of this study include potential influences in causal inference by biological pleiotropy, assortative mating, and the nonrandom sampling of study subjects.

**Conclusions:**

Our results demonstrate that the observed association between maternal height and fetal growth measures (i.e., birth length and birth weight) is mainly defined by fetal genetics. In contrast, the association between maternal height and gestational age is more likely to be causal. In addition, our approach that utilizes the genetic score derived from the nontransmitted maternal haplotype as a genetic instrument is a novel extension to the Mendelian randomization methodology in casual inference between parental phenotype (or exposure) and outcomes in offspring.

## Abstract 2

Using a Mendelian randomization approach, Ge Zhang and colleagues examine the causal relationship between maternal height, birth size, and gestational age at birth.

## Editors' Summary

**Background:**

Soon after the birth of a baby, doting parents send messages to friends and relatives or post information on social media sites to let everyone know when their new baby boy or girl was born. They may also post information about how heavy he/she was at birth and his/her length. These pregnancy outcomes, together with gestational age at birth (the length of time that a baby has spent developing in its mother’s womb), affect the baby’s immediate health and survival. Importantly, however, these pregnancy outcomes are also associated with the risk of long-term adverse health outcomes such as obesity, cardiometabolic disorders (heart disease and conditions such as diabetes that affect how the body makes energy from food), and neuropsychiatric conditions (mental disorders attributable to diseases of the nervous system, such as depression). For example, some studies have shown an association between low birth weight and an increased risk of type 2 diabetes later in life.

**Why Was This Study Done?:**

Identification of the environmental and genetic factors that causally influence gestational age, length, and weight at birth would improve our understanding of why these pregnancy outcomes are associated with disease during adulthood and could help in the design of strategies to prevent these diseases. Epidemiological studies (investigations that examine disease patterns in populations) suggest that, compared to tall mothers, short mothers tend to deliver their babies at earlier gestational ages, with lower birth weights and lengths. Epidemiological studies cannot show, however, whether variations in maternal height cause variations in pregnancy outcomes. Other characteristics shared by tall mothers might actually determine the size and gestational age of their offspring (confounding). Here, the researchers use “Mendelian randomization” to assess the causal effect of maternal height on the size and gestational age at birth of babies. Because gene variants are inherited randomly, they are not prone to confounding. So, if maternal height actually affects gestational age and size at birth, genetic variants (instruments) that affect maternal height should be associated with differences in gestational age and size at birth, provided confounding due to the transmission of parental alleles (variant forms of genes; people have two alleles of every gene, one inherited from each parent) is avoided by adjusting for the baby’s genotype.

**What Did the Researchers Do and Find?:**

The researchers used phenotype data (observable characteristics such as maternal height and birth weight of the baby) and single nucleotide polymorphism (SNP; a type of genetic variant) data obtained from 3,486 Nordic mother/baby pairs. Analysis of the phenotype data indicated that maternal height was significantly associated with length, weight, and gestational age at birth (a significant association is unlikely to have arisen by chance). For their Mendelian randomization analysis, the researchers constructed a genetic score based on 697 SNPs known to be associated with adult height. To avoid confounding due to genetic sharing between the mother and baby, they determined which of the height-associated alleles each baby had inherited from its mother and used the nontransmitted haplotype score as a genetic instrument for maternal height (a haplotype is a set of DNA variations that are inherited together). Birth length and weight were significantly associated with both the maternal and paternal transmitted haplotype scores but not with the maternal nontransmitted haplotype score. However, gestational age was significantly but modestly associated with the maternal nontransmitted haplotype score.

**What Do These Findings Mean?:**

The validity of the assumptions that underlie the Mendelian randomization approach and the design of the studies supplying the data for this analysis may affect the accuracy of the findings reported here. Nevertheless, these findings suggest that the observed association between maternal height and fetal growth measurements is mainly determined by the genetics of the baby. That is, differences in maternal height do not cause differences in birth weight or length. Rather, some of the gene variants that the baby inherits from its mother determine both its size and its mother’s height. These findings also provide weak evidence that the association between maternal height and gestational age is causal. Maternal height might, for example, causally influence gestational age by limiting the space available for the baby’s growth before birth. Finally, these findings introduce an extension to the Mendelian randomization approach that can be used to investigate causal associations between parental characteristics and offspring outcomes.

**Additional Information:**

This list of resources contains links that can be accessed when viewing the PDF on a device or via the online version of the article at http://dx.doi.org/10.1371/journal.pmed.1001865.

- The March of Dimes, a not-for-profit organization for pregnancy and baby health, provides information about low birth weight and its consequences
- Nemours, a not-for-profit organization for child health, provides information about the weight of newborn babies (in English and Spanish)
- Wikipedia has pages on birth weight, gestational age, and Mendelian randomization (note: Wikipedia is a free online encyclopedia that anyone can edit; available in several languages)
- MedlinePlus provides information and links to additional resources about birth weight and a brief explanation of gestational age (in English and Spanish)

## Introduction

Gestational age at birth (or simply gestational age), birth length, and birth weight are pregnancy outcomes associated not only with perinatal morbidity and mortality but also with long-term adverse health outcomes, such as obesity [1], cardiometabolic disorders [2,3], and neuropsychiatric conditions [4]. Identifying the genetic and environmental factors that causally influence these pregnancy outcomes will increase the understanding of the mechanisms underlying their association with diseases that occur later in life and thereby provide clues for possible prevention strategies [5,6].

Observational epidemiological studies have revealed several factors that are associated with birth length, birth weight, and gestational age. Examples include maternal age, height, and weight, offspring sex [7], maternal exposure to environmental hazards [8], and physiological or psychological stresses [9]. In addition, genetic factors in both mothers and infants influence these pregnancy outcomes [6,10,11]. Among these factors, maternal height has been repeatedly reported to associate with fetal birth length [12], birth weight [7,13], and gestational age [14–18]. Associations between paternal height and fetal birth length [19,20] as well as birth weight [21,22] have also been reported, although there was no association with gestational age [15].

**Fig 1:**
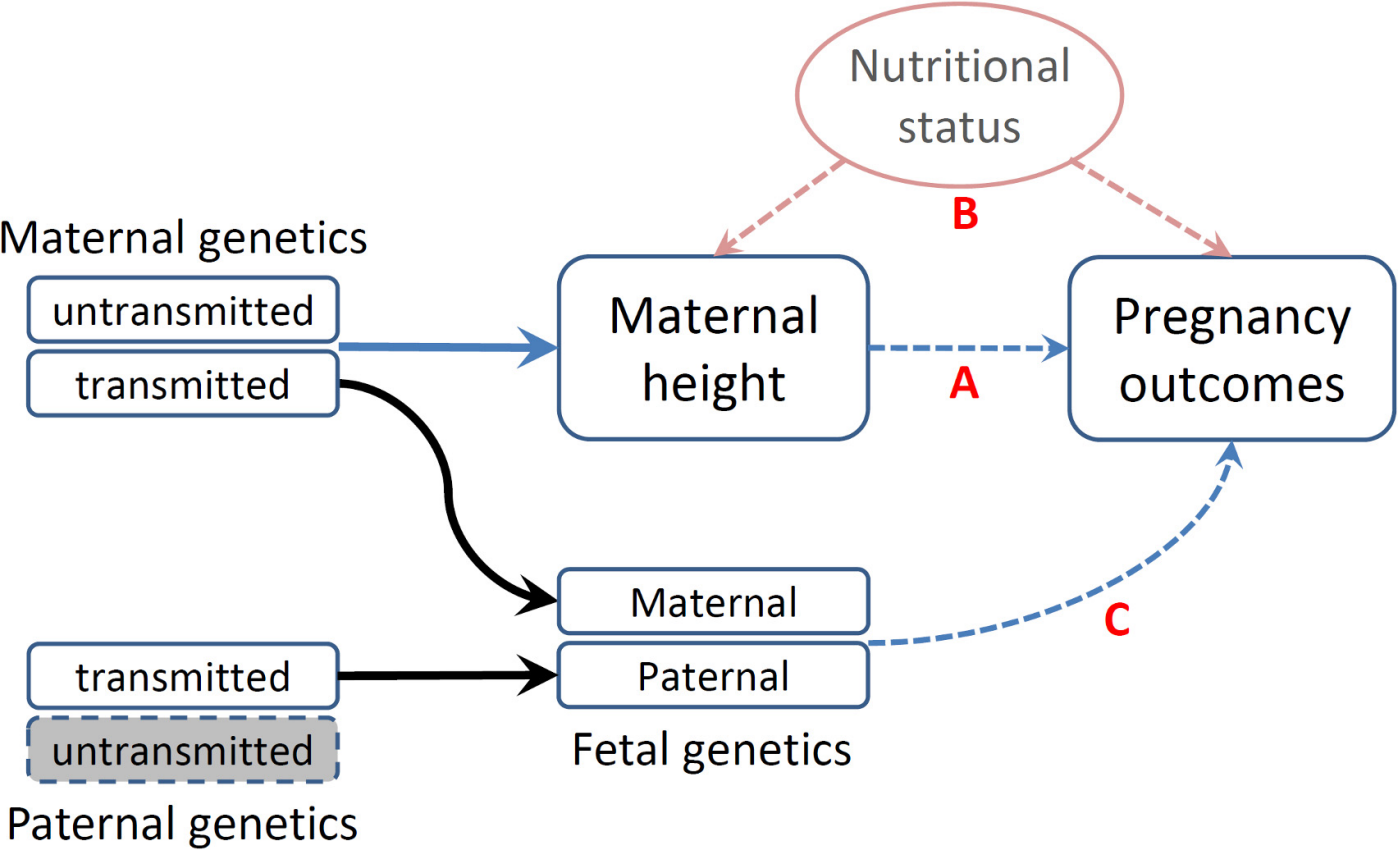
Schematic representation of various causal mechanisms that can lead to the observational associations between maternal height and pregnancy outcomes. (A) Direct causal effect of maternal height on pregnancy outcomes. (B) Associations of social and nutritional confounders that have impacts on both maternal height and pregnancy outcomes. (C) Fetal genetics that directly influences pregnancy outcomes.

The associations between maternal height and pregnancy outcomes have been interpreted based upon a mechanistic assumption—that maternal height sets a physical constraint on the intrauterine environment (shorter women may have a small uterus size, limiting fetal growth) (Fig 1A—direct causal effect) [23]. Furthermore, adult height may reflect a mother’s cumulative social and nutritional condition over her life course being an indicator of the persisting biological and/or environmental factors that impact her offspring’s growth in utero (Fig 1B—indirect association) [24]. The latter could be particularly important in low- and middle- income countries, where nutrition-related factors may substantially restrict one’s growth [25]. These effects—physical constraints or confounding nutritional factors that jointly influence the fetal growth environment—are commonly referred to as environmental effects (intrauterine effects) [26]. In addition to these nongenetic mechanisms, the association between maternal height and birth outcomes could be attributed to genetics in that the genetic polymorphisms that influence maternal height may also have direct functional effects on pregnancy outcomes in fetus (Fig 1C—genetic effect) [6,11,20,27,28].

Disentangling these different mechanisms underlying the association between maternal height and pregnancy outcomes is important, as the knowledge may enhance our understanding of the genetic and environmental etiology of these important pregnancy outcomes and how they impact health. However, only a few study designs are capable of discerning these different mechanisms and drawing causal inferences with regard to the association between maternal height and pregnancy outcomes [26]. The existing literature on the genetic and environmental impacts on pregnancy outcomes is inconsistent: some studies suggest that intrauterine factors have the major role on birth weight [29], and some studies suggest that maternal height is mainly a proxy for maternal socioeconomic conditions [24,30], while some studies suggest that genetic factors have the major influence on pregnancy outcomes [20,28].

One approach to distinguish the contributions of intrauterine environmental effects from direct genetic influences is to compare maternal–offspring and paternal–offspring associations. Stronger maternal–offspring than paternal–offspring associations would imply intrauterine effects, whereas similar maternal and paternal associations with a birth outcome would suggest predominant genetic effects [31,32]. Previous studies have indicated that both maternal and paternal height are associated with birth length [20] and birth weight [22,28], which suggest substantial fetal genetic impact on early fetal growth. In contrast, gestational age was reported to be associated with maternal height but not with paternal height [15].

More recently, Eaves et al. [33] extended the genome-wide complex trait analysis (GCTA) method to partition offspring phenotypic variance into components attributable to fetal genotype, maternal effects, and the covariance between these two. By applying this method to the Avon Longitudinal Study of Parents and Children (ALSPAC) cohort, they found that fetal genetics and maternal effect explain 13% and 11%, respectively, of the variance in birth length [33].

Another approach to draw causal inference is Mendelian randomization [34,35], which utilizes genetic variants associated with the parental phenotype/exposure as an instrumental variable to probe the causal effect of parental phenotype/exposure on outcomes in their offspring [26,36]. As an “instrumental variable” technique [37], the validity of this method depends on two conditions: (1) relevance—the genetic instrument must be associated with the intermediate phenotype or risk factor with enough strength—and (2) exogeneity—the genetic instrument must not be related to the outcome by means other than the intermediate phenotype. Specifically, the latter assumption requires that the genetic instrument is unrelated to confounders and that the genetic instrument does not influence the outcome through other mechanisms (biological pleiotropy) [38].

**Box 1. Assumptions of Mendelian Randomization Study**

Mendelian randomization is a type of instrumental variable analysis that uses a genetic instrumental variable (*G*) to examine the causal effect of an exposure or an intermediate phenotype (*X*) on an outcome (*Y*). In our study, we used the height genetic score as the genetic instrument (*G*) to probe the causal relationship between maternal height (*X*) and various pregnancy outcomes (*Y*). The validity of this approach requires the following assumptions to be met:

1. *G* is associated with *X* with enough strength, which is called the instrument relevance in statistical terms.
2. *G* is related to *Y* only through *X*, not through other environmental confounding factors or biological pleiotropy. This assumption is also referred to as instrument exogeneity.

In this study, the first assumption was achieved by using a genetic score constructed based on a large number of height-associated SNPs. The genetic score explains a substantial fraction of variance in maternal height and therefore makes a powerful genetic instrument. Because *G* is randomly assigned to individuals at birth, *G* is generally not associated with nongenetic confounders. However, it is hard to completely exclude the possibility of biological pleiotropy, i.e., *G* may influence *Y* through biological mechanisms other than *X*.

In the inference of a causal relationship between a parental phenotype and an outcome in offspring, parental genotype is an invalid instrument if the offspring genotype is not appropriately adjusted for, because half of the parental alleles are transmitted to offspring and may directly influence the outcome in offspring. To overcome this problem, we used a genetic score based on the nontransmitted haplotype to index the maternal height.

Since genetic variants typically explain a small proportion of the phenotypic variance, conventional Mendelian randomization studies often require large sample sizes [39]. Using height as an example, the strongest single SNP association only explains <0.5% of the observed variation in height [40,41]. Thus, a Mendelian randomization study using a single SNP would require hundreds of thousands of samples [42]. One solution to this limitation is to use multiple associated SNPs obtained from large-scale genome-wide association studies (GWAS) to construct more powerful genetic instruments [43–45].

When utilizing the Mendelian randomization to draw causal inference between parental phenotype and outcomes in offspring, the method is further complicated by the transmission of parental alleles, and therefore, the fetal genotype needs to be adjusted for to avoid the confounding due to genetic sharing between parents and offspring [26,35]. Using this method, Lawlor et al. [46] explored the causal effect of maternal body-mass index (BMI) on childhood obesity. We have not seen this approach being used to examine the causal mechanism underlying the association between maternal height and pregnancy outcomes.

In this study, we first examined the observational associations between maternal height and birth length, birth weight, and gestational age at birth and then assessed the causal effect of maternal height on these measurements by a Mendelian randomization approach. We constructed genetic score based on 697 height-associated SNPs as the instrumental variable for maternal height. We inferred parental transmission of the height-associated SNPs and utilized the haplotype genetic score derived from the nontransmitted alleles as a valid genetic instrument and thus avoided the interference by genetic transmission in causal inference. Using this modified Mendelian randomization approach, we evaluated the causal relationship of maternal height on birth size and gestational age at birth in 3,485 mother/infant pairs collected from three Nordic countries (Finland, Denmark, and Norway).

## Methods

### Description of Study Cohorts

We used the phenotype and genome-wide SNP data of up to 3,485 mother/infant pairs from birth cohorts collected from three Nordic countries (Table 1).

**Table 1:** Description of study cohorts.

Cohort	Full Name	Mother/Infant Pairs (Term/Preterm)^*^	Genotyping Platform
FIN	Finnish (Helsinki) Birth Cohort	544/239	Affymetrix 6.0 + Illumina
MoBa	Norwegian Mother and Child Cohort	525/493	Illumina Human660W
DNBC	Danish National Birth Cohort	960/724	Illumina Human660W

* Preterm birth was defined as gestational age less than 37 wk; term birth was defined as gestational age larger than 38 wk in this study.

The Finnish cohort (FIN) was collected for a genetic study of preterm birth [47]. Briefly, whole blood samples were collected from ~800 mother/child pairs from the Helsinki (southern Finland) University Hospitals between 2004 and 2014. All of these studied samples are of Finnish descent. Crown-rump length at the first ultrasound screening between 10+ and 13 wk was used to determine the gestation and the due date. The study was approved by the Ethics Committee of Oulu University Hospital and that of Helsinki University Central Hospital. Written informed consent was given by all participants.

The Mother Child Cohort of Norway (MoBa) is a nationwide Norwegian pregnancy cohort study administered by the Norwegian Institute of Public Health. The study includes more than 107,000 pregnancies recruited from 1999 through 2008. Gestational age was estimated by ultrasound at gestational weeks 17–19. In the few participants without ultrasound dating, gestational age was estimated using the date of the last menstrual period. For the current study, we used the mother-child pairs that were selected from version 4 of the MoBa cohort, which included a total of 71,669 pregnancies. Singleton live-born spontaneous pregnancies with mothers in the age group 20–34 y were selected. Pregnancies involving preexisting medical conditions and pregnancies with complications, as well as pregnancies conceived by in vitro fertilization, were excluded from the study. Random sampling was done from two gestational age ranges of 154–258 d (cases) and 273–286 d (controls). In total, 3,121 mothers and children were genotyped [48]. Of the 2,977 samples that passed quality control (QC), 1,018 mother/child pairs were identified and used in this study. All parents gave informed, written consent. The study was approved by the Regional Committee for Medical Research Ethics South-East, Norway.

The Danish National Birth Cohort (DNBC) followed over 100,000 pregnancies between 1997 and 2002, with extensive epidemiologic data recorded on health outcomes in both mother and child [49]. The current study used the data downloaded from the Database of Genotypes and Phenotypes (dbGaP) (http://www.ncbi.nlm.nih.gov/projects/gap/cgi-bin/study.cgi?study_id=phs000103.v1.p1), which contains data from a genome-wide case/control study using approximately 1,000 preterm mother-child pairs (gestational age between 22–37 wk) from the DNBC, most with spontaneous onset of labor or preterm premature rupture of membranes (PPROM), along with 1,000 control pairs in which the child was born at ~40 wk gestation. Gestational age in this cohort was estimated by combining all available information from multiple sources: self-reported date of last menstrual period, self-reported delivery date, and gestational age at birth registered in the Medical Birth Register and the National Patient Register. Of the 3,838 DNBC samples with genotype data, 3,712 samples passed the genotype QC, from which we identified 1,684 mother/child pairs and used them in the current study.

All the preterm births included in this study were spontaneous. Obstetrical induction of labor, placental abnormalities, preeclampsia, congenital malformations, and multiple births were excluded. Pregnancies involving preexisting medical conditions known to be associated with preterm birth and pregnancies with complications were also excluded.

### Genotyping and Imputation

Genome-wide SNP genotyping was conducted using different SNP arrays (Table 1). Specifically, for FIN, genotyping was conducted using Affymetrix 6.0 (Affymetrix, California, United States) and various other Illumina arrays (Illumina, California, United States). For the Affymetrix SNP Array 6.0, genotype calls were determined using the CRLMM algorithm [50,51] among chips that passed the vendor-suggested QC (Contrast QC > 0.4). For the Illumina chips, the genotype calling was conducted using Illumina’s genotyping module v1.94 in the GenomeStudio v2011.1. The 3,121 samples from the MoBa cohort were genotyped using the Illumina Human660W-Quadv1_A bead chip (Illumina), and the genotype calls were determined using CRLMM algorithm. The DNBC samples were also genotyped using the Illumina Human660W-Quadv1_A bead chip, and the genotype calls were determined using the CRLMM algorithm based on the chip intensity files (IDAT files) obtained from dbGaP.

Similar QC procedures were used across the three studies. Briefly, individuals with incorrect sex assignment, excessive heterozygosity, or low call rate (<98%) were excluded from further analysis. Incorrect mother-child relationships and cryptic relatedness were detected by identity-by-descent (IBD) sharing estimated from genome-wide SNPs. Non-European samples were identified and excluded using principal component analysis (PCA) anchored with 1000 Genomes or HapMap reference samples. At marker level, SNPs with low call rate (<98%), low minor allele frequency (<0.03), or significant deviation from Hardy-Weinberg equilibrium (*p* < 1 × 10^−4^) were excluded.

Following the QC procedures, we conducted genome-wide imputation using the reference haplotype data extracted from the 1000 Genomes Project (integrated phase I release) [52]. A standard two-step imputation procedure was followed: the genotype data was first prephased using Shapeit2 software [53], and then the estimated haplotypes were used to impute untyped SNPs in mothers and infants using the reference haplotypes extracted from the 1000 Genomes Project (integrated phase I release) by either Minimac [54] (FIN) or Impute2 [55] (MoBa and DNBC).

### Genetic Score Analysis

We selected the 697 SNPs associated with adult height from the stage II meta-analysis [40] of GIANT studies (http://www.broadinstitute.org/collaboration/giant) to construct a weighted genetic score and use it as a genetic instrument of height. The list of height-associated SNPs and their effect size estimates were extracted from the supplementary table of reference [40]. The reported effect sizes were estimated using an approximate conditional and joint analysis, which appropriately accounts for the linkage disequilibrium (LD) between SNPs [56]. The weighted genetic score was constructed as *S* = ∑*b*_*i*_ *G*_*i*_, where *G*_*i*_ = 0, 1 or 2 (for genotype-based genetic score) or *G*_*i*_ = 0 *or* 1 (for haplotype-based genetic score, see below), which indicates the number of reference alleles for a specific SNP, and *b*_*i*_ is the estimated allelic effect.

### Determining Parental Origin

**Fig 2:**
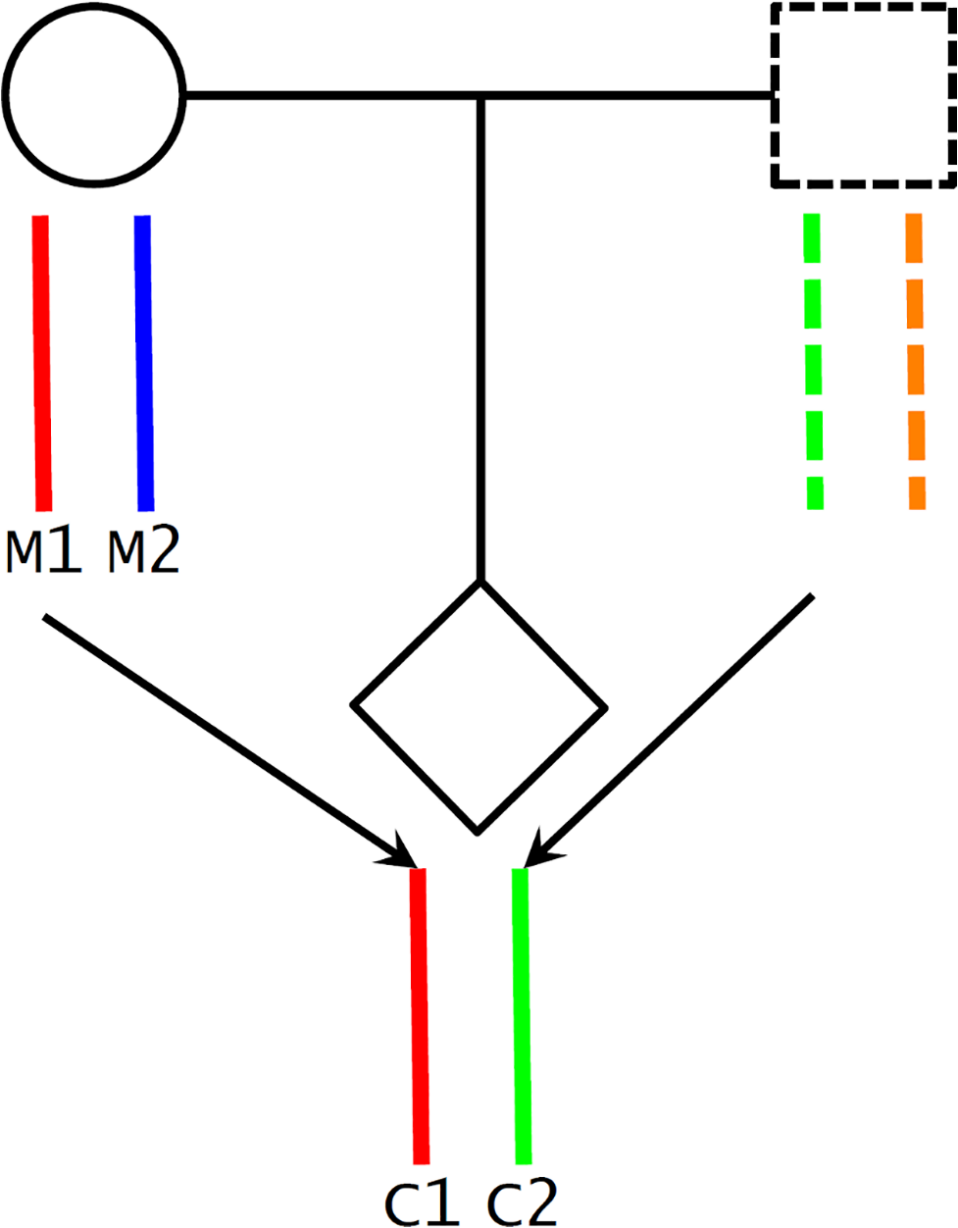
Maternal transmission of alleles and haplotype scores. We inferred maternal transmission of alleles (M1 → C1) and constructed haplotype scores: M1 and C1, maternal transmitted haplotype score; M2, maternal nontransmitted haplotype score; and C2, paternal transmitted haplotype score.

A potential challenge in utilizing Mendelian randomization to study causal effects of parental exposures on outcomes in offspring is the confounding due to shared genetics between parents and offspring [26,46]. Using our case as an example, maternal genotype-based genetic score (parental genotype) is not a valid instrument for maternal height (parental phenotype) because the transmitted maternal alleles can influence the fetal phenotypes (e.g., birth length or weight) through direct genetic effects in the infants (Fig 1C). To overcome this problem, we inferred maternal transmission of height-associated alleles and utilized the nontransmitted haplotype genetic score as a genetic instrument to avoid the interference by genetic transmission (Fig 2). Specifically, for a particular SNP, we inferred allelic transmission (from mother to child) based on either direct comparison of genotypes or local haplotype sharing. If either or both of mother and child are homozygotes, the allele transmission can be unambiguously determined from their genotypes. When both mother and child were heterozygotes, we constructed a long-range (up to 1 Mb) local haplotype around the SNP under consideration and compared haplotype sharing to determine allelic transmission. With the inferred allelic transmission of the height-associated SNPs, we divided the maternal genetic score into two haplotype components (Fig 2): “maternal transmitted haplotype score” (M1), which was calculated from the transmitted alleles, and “maternal nontransmitted haplotype score” (M2), which was calculated from the nontransmitted alleles in the mothers. Accordingly, the fetal genetic score was divided into “maternal transmitted haplotype score” (C1) and “paternal transmitted haplotype score” (C2). For example, if we use *A*_*i*_ and *B*_*i*_ to denote the transmitted and nontransmitted alleles of SNP *i* in a mother, the maternal transmitted haplotype score (M1) was calculated as ∑*b*_*i*_ *A*_*i*_ and the maternal nontransmitted haplotype score (M2) was ∑*b*_*i*_ *B*_*i*_. Obviously, the genotype score equals ∑*b*_*i*_ *G*_*i*_ = ∑*b*_*i*_ *A*_*i*_ + ∑*b*_*i*_ *B*_*i*_, since the genotype is the summation of the two alleles (*G*_*i*_ = *A*_*i*_ + *B*_*i*_). The two fetal haplotype scores (C1 and C2) were calculated in the same way, and C1 = M1. Association between the maternal transmitted haplotype score (M1/C1) and a fetal phenotype can be explained as either intrauterine effects (Fig 1A) or direct genetic influence of fetal genetics (Fig 1C), since the transmitted alleles can have genetic influence in both mothers and infants. Whereas the association between the maternal nontransmitted haplotype score (M2) and a fetal phenotype is a clear indication of causal effects of the maternal phenotype itself (Fig 1A), the association between the paternal transmitted haplotype score (C2) and a fetal phenotype should represent fetal genetic effect (Fig 1C).

### Statistical Analyses

We first assessed statistical associations between maternal phenotypes (i.e., height, weight, BMI, and age) and pregnancy outcomes (i.e., birth length, birth weight, and gestational age) using linear regression. Associations between various height genetic scores and maternal phenotype as well as pregnancy outcomes were examined by regression analysis. In the regression analyses of birth length and birth weight, maternal age, fetal gender, and gestational age were included as covariates. In the analysis of gestational age, only maternal age and fetal gender were included. To estimate the magnitude of causal effects of maternal height on pregnancy outcomes, we performed instrumental variable analysis using the two-stage least squares approach [57]. Three genetic instrumental variables for maternal height were evaluated: (1) maternal genotype-based genetic score without controlling for fetal genetic score (method 1), (2) maternal genotype-based genetic score while controlling for fetal genetic score (method 2), and (3) maternal nontransmitted haplotype score (method 3). Fixed-effect meta-analysis was used to combine the results from different studies. Between-study heterogeneity was tested by Cochran’s Q test.

## Results

### Association between Maternal Height and Pregnancy Outcomes

Summary statistics of the phenotype data of the three cohorts are provided in S1 Table and S2 Table. We observed significant associations between maternal height and birth weight, birth length, and gestational age at birth separately in all the three studied cohorts (Table 2). The meta-analysis *p*-values were 6.31 × 10^−9^ (birth length), 2.19 × 10^−15^ (birth weight) and 1.51 × 10^−7^ (gestational age).

**Table 2:** Association between maternal height and pregnancy outcomes.

Cohort	Birth Length			Birth Weight			Gestational Age		
	beta^*^	se	*p*-value	beta	se	*p*-value	beta	se	*p*-value
FIN	0.0536	0.0113	2.29E-06	11.3	2.42	3.48E-06	0.293	0.142	0.0393
MoBa	0.0379	0.0109	0.00054	6.31	2.01	0.00171	0.303	0.104	0.0035
DNBC^$^				11.1	1.88	4.12E-09	0.400	0.102	9.34E-05
									
meta	0.0455	0.00783	6.31E-09	9.46	1.19	2.19E-15	0.340	0.0647	1.51E-07
p_het^#^	0.317			0.149			0.746		

* Beta is the unstandardized coefficient, which shows the estimated changes in pregnancy outcomes per 1 cm increase in maternal height.

$ DNBC does not have birth-length data.

# p_het: heterogeneity test *p*-value.

### Association between Maternal Height Genetic Score and Maternal Height

As expected, the maternal genetic score was significantly associated with maternal height (meta *p* = 2.75 × 10^−189^) and could explain a large fraction (~20%) of the observed variance in maternal height in all of the three cohorts (Table 3). The transmitted haplotype score and the nontransmitted haplotype scores explained similar proportions of variance (~10%) (S3 Table). At single SNP level, the estimated effect sizes (on maternal height) of the height-associated SNPs based on our studies were highly correlated with the reference study (Pearson’s ρ = 0.41, *p* < 2 × 10^−16^).

**Table 3:** Association between maternal height genetic score and maternal height.

Cohort	Maternal Genotype Genetic Score			
	Beta	se	*p*-value	r2
FIN	5.67	0.397	3.48E-41	0.209
MoBa	5.54	0.347	3.82E-51	0.207
DNBC	5.80	0.289	3.62E-80	0.202
meta	5.69	0.194	2.75E-189	
p_het	0.841			

### Association between Maternal as well as Fetal Height Genetic Scores and Pregnancy Outcomes

The maternal genetic score showed consistent significant associations with both birth length (meta *p* = 3.04 × 10^−5^) and birth weight (meta *p* = 1.19 × 10^−7^) in the studied cohorts. The fetal height genetic score was even more significantly associated with birth length (meta *p* = 5.40 × 10^−9^) and birth weight (meta *p* = 1.84 × 10^−12^), which indicated direct fetal genetic influence of height-associated SNPs on fetal growth. After adjustment for fetal genetic score, the associations between maternal genetic score and birth length or birth weight were no longer significant (Table 4 and S4 Table).

**Table 4:** Association between genotype height genetic scores and pregnancy outcomes based on meta-analysis.

Genotype Score	Birth Length			Birth Weight			Gestational Age		
	beta	se	*p*-value	beta	se	*p*-value	beta	se	*p*-value
Maternal	0.382	0.0916	3.04E-05	75.4	14.2	1.19E-07	1.34	0.760	0.0769
Fetal	0.535	0.0916	5.40E-09	101	14.3	1.84E-12	0.349	0.769	0.650
Maternal (Adjusted)^*^	0.135	0.108	0.214	30.6	16.8	0.0697	1.62	0.902	0.0717

* Adjusted by fetal genetic score.

Given the highly significant association between fetal genetic score based on adult height-associated SNPs with both birth length and birth weight, we further examined single SNP associations of these adult height-associated SNPs with both birth length and birth weight in the infants. As indicated by the quantile-quantile (Q-Q) plots (S1 Fig), there was a general inflation of the association test statistics (λ = 1.137 and 1.175 for birth length and weight, respectively), indicating that a substantial fraction of the height-associated SNPs were also associated with birth length or birth weight in fetus. There was also a marginally significant correlation between the estimated effect size of these SNPs on birth length and the reported effect size on adult height (Pearson’s ρ = 0.073, *p* = 0.055). However, the correlation of the estimated effect size of these SNPs on birth weight and the reported effect size on adult height was not significant (Pearson’s ρ = 0.041, *p* = 0.283).

**Fig 3:**
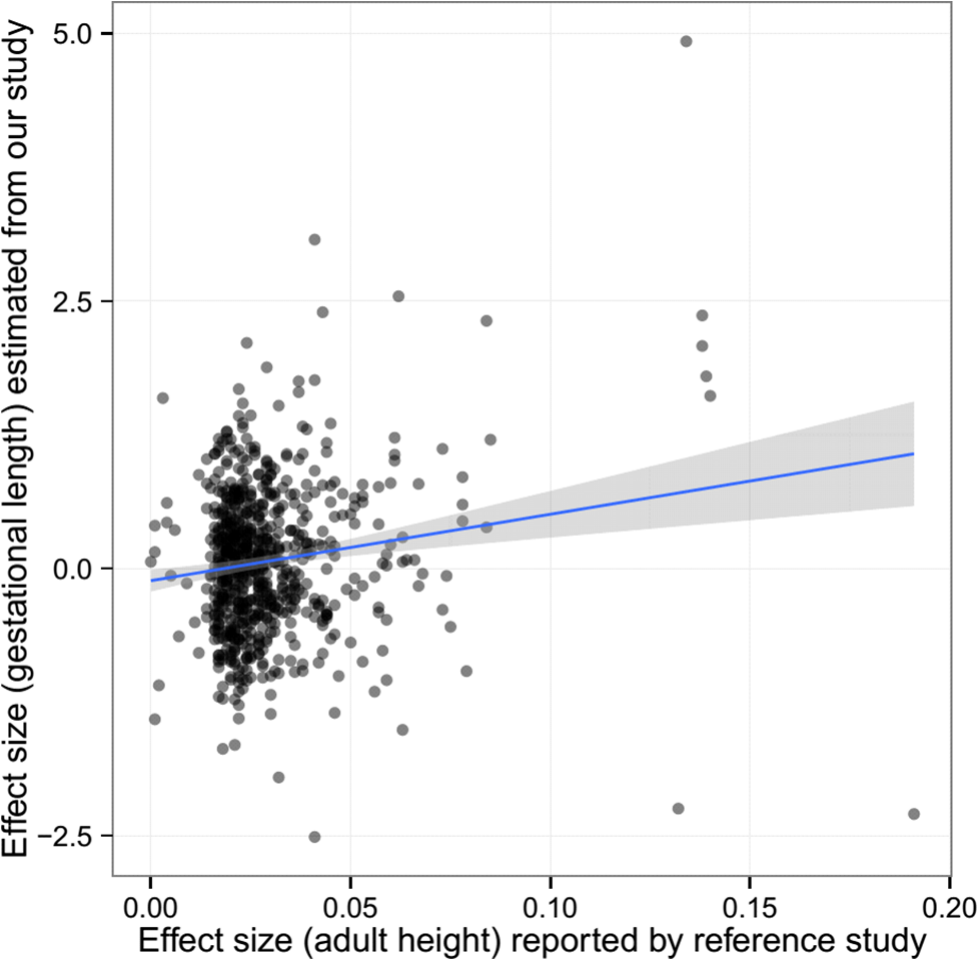
The estimated effect (in mothers) of the height-associated SNPs on gestational age was correlated with reported effect size on adult height.

The pattern of associations for gestational age was different from those observed in birth length or birth weight. There was some evidence of association between maternal height genetic score and gestational age overall (meta-analysis *p* = 0.077), preferably in the DNBC cohort (*p* = 0.047). However, the fetal height genetic score was not significantly associated with gestational age. After adjustment for fetal genetic score, the maternal height genetic score was still marginally associated with gestational age (meta *p* = 0.072). Further single marker association (between maternal genotype and gestational age) analysis also showed some inflation of association test statistics (λ = 1.054, S2 Fig) and a significant correlation between the estimated effect size of these SNPs on gestational age and the reported effect size on adult height (Pearson’s ρ = 0.152, *p* = 5.58 × 10^−5^) (Fig 3), indicating the effect of these height-associated SNPs on gestational age is projected through a common intermediate phenotype (i.e., height) rather than different biological (pleiotropic) mechanisms.

### Association between Haplotype Height Genetic Scores and Pregnancy Outcomes

Our parental haplotype genetic score analysis revealed significant associations of both maternal transmitted haplotype score and paternal transmitted haplotype score with birth length and birth weight (Table 5 and S5 Table). However, the associations between the maternal nontransmitted haplotype scores with these two fetal growth traits were far less significant (meta *p* = 0.0405 and 0.404 for birth length and birth weight, respectively). In addition, although both were significantly associated with birth weight, the effect of maternally transmitted alleles was significantly larger than paternally transmitted alleles (*p* = 0.0053). This difference of effect was not observed for birth length. In contrast, the maternal nontransmitted haplotype score (M2), but not the parental transmitted score (C2), showed significant associations with gestational age (meta *p* = 0.0424), which suggests cross-generational effect of maternal height SNPs on gestational age through an intermediate phenotype (maternal height) but not through genetic inheritance.

**Table 5:** Association between haplotype genetic scores and pregnancy outcomes.

Haplotype Score^*^	Birth Length			Birth Weight			Gestational Age		
	beta	se	*p*-value	beta	se	*p*-value	beta	se	*p*-value
M1 (C1)	0.534	0.136	8.08E-05	145	20.9	4.02E-12	0.587	1.12	0.601
M2	0.270	0.132	0.0405	17.1	20.4	0.404	2.204	1.09	0.0424
C2	0.557	0.129	1.61E-05	63.8	20.3	0.00165	0.203	1.08	0.851

* M1 (C1), maternal transmitted haplotype score; M2, maternal nontransmitted haplotype score; C2, paternal transmitted haplotype score.

Although not a primary objective of this current study, we also examined the pairwise correlations between the various genetic scores (Table 6 and S6 Table). In addition to those highly significant correlations due to genetic sharing between parents and their offspring, such as correlations between maternal and fetal genetic scores, we also observed significant positive correlations between the two maternal haplotype genetic scores (transmitted and nontransmitted scores) and between the paternally transmitted score and the maternal scores, which were most probably due to assortative mating based on height.

**Table 6:** Pairwise correlation among genetic scores.

Score^*^	M	M1	M2	C	C2
M					
M1 (C1)	0.715 (0)				
M2	0.734 (0)	0.051 (0.00128)			
C	0.539 (8.63e-277)	0.704 (0)	0.086 (1.66e-07)		
C2	0.067 (3.41e-05)	0.024 (0.0770)	0.073 (7.87e-06)	0.726 (0)	

* M, maternal genotype score (M1 + M2); M1 (C1), maternal transmitted haplotype score; M2, maternal nontransmitted haplotype score; C, fetal genotype score (C1 + C2); C2, paternal transmitted haplotype score.

### Instrumental Variable Analysis

To estimate the causal effect of maternal height on the pregnancy outcomes, we performed instrumental variable analysis (Table 7 and S7 Table). For comparison purposes, we tested three different methods using either maternal genotype-based genetic score (method 1 and 2) or maternal nontransmitted haplotype score (method 3) as instrument variables. For birth length and birth weight, the causal inference based on method 1 (using maternal genotype-based genetic score without controlling for fetal genetic score) were highly significant. However, the significance diminished after controlling for fetal genetic score (method 2) or when using maternal nontransmitted haplotype score as the genetic instrument (method 3). Consistent with our genetic score association analyses, these results again suggested that fetal genetic effect instead of causal effect of maternal height itself was the main driving force behind the observed associations between maternal height and fetal growth measures. Although the inference based on the maternal nontransmitted haplotype score (method 3) indicated some significant causal influence of maternal height on birth length (*p* = 0.0336). In contrast, all three methods revealed consistent and significant causal effect of maternal height on gestational age, with every 1 cm increase in maternal height resulting in ~0.4 more gestational d. To exclude the possible bias introduced by assortative mating (i.e., due to the correlation between haplotype scores), we performed instrumental variable analysis with maternal and paternal transmitted scores (M1/C1 and C2) adjusted as covariates (method 4). The results were similar to those obtained by method 3.

**Table 7:** Results of instrumental variable analysis.

Method^*^	Birth Length			Birth Weight			Gestational Age		
	beta	se	*p*-value	beta	se	*p*-value	Beta	se	*p*-value
Method 1	0.0692	0.0161	1.64E-05	13.4	2.53	1.21E-07	0.280	0.135	0.0385
Method 2	0.0298	0.0214	0.164	5.70	3.30	0.0837	0.387	0.176	0.0276
Method 3	0.0501	0.0236	0.0336	2.47	3.70	0.504	0.440	0.194	0.0234
Method 4	0.0418	0.0257	0.104	0.486	3.96	0.170	0.475	0.209	0.0232

* Method 1 and method 2 use maternal genotype-based genetic score (M) as the instrument variable for maternal height without (method 1) or with (method 2) controlling for fetal genetic score (C). Method 3 utilizes maternal nontransmitted haplotype score (M2) as a valid instrument for maternal height. Method 4 uses maternal nontransmitted haplotype score (M2) as the instrument variable and further adjusts for possible bias due to assortative mating by controlling for maternal and paternal transmitted scores (M1/C1 and C2) as covariates.

## Discussion

### Summary of Major Findings

While the extremes of birth size, particularly birth weight, and gestational age at birth are important global contributors to infant mortality, more modest deviations in these parameters have been repeatedly linked to risk for adult disorders such as type 2 diabetes mellitus, hypertension, and cardiovascular disease [58–60]. Thus, understanding the determinants of fetal growth and the duration of pregnancy are essential to improve both newborn and later-in-life health. Epidemiological evidence has suggested that these birth outcomes are shaped by intrauterine environment, but the relative contribution of maternal genetic, fetal genetic, and nongenetic factors that may drive epigenetic physiological adaptations remains incompletely understood. Moreover, should fetal genetics shape these outcomes, their direct involvement in the risk for the adult diseases, as well, is an intriguing possibility. Recently, to better understand normal fetal growth in healthy pregnancy, a multinational longitudinal assessment has been reported [61]. These data demonstrate similar average growth profiles across geographic populations but do not define the mechanisms for variability within a given population.

In this study, we observed the consistent associations between maternal height and birth length, birth weight, and gestational age in each of the three study cohorts. The estimated effect sizes of the observed associations were respectively ~0.05 cm/cm for birth length, ~10 g/cm for birth weight, and ~0.34 d/cm for gestational age per 1 cm change in maternal height, which are in the same range as previous reports for fetal growth [20] and gestational age [15]. We then disentangled causal relationships underlying these observational associations.

Based on an extensive list of height-associated SNPs (*n* = 697) reported by a recent large GWA meta-analysis [40], we constructed a height genetic score as a powerful genetic instrument for maternal height. In our study samples, the genotype-based genetic score explained a substantial fraction (~20%) of variance in maternal height. This percentage was even larger than that (~16%) reported in the original study [40], which is likely due to the homogeneity of our samples (i.e., all females with northern European origin and a more restricted age distribution). Our previous studies have shown that the height genetic score explained larger variance in females than males [62,63].

We observed significant associations of maternal height genetic score with birth length, with birth weight, and, to a lesser extent, with gestational age. As the genetic score captures the genetic variation in maternal height, these significant associations suggested either direct causal influence (Fig 1A) of maternal height or fetal genetic effect (Fig 1C) rather than confounding effects (Fig 1B), although the latter mechanism could not be excluded. It should be emphasized that maternal genetic score is not a valid instrument for maternal height, unless adjusted for fetal genotype; thus, it cannot be used to differentiate the causal effect of maternal height (Fig 1A) from fetal genetic effect (Fig 1C).

We further probed the causal impacts of maternal height using genetic instruments either based on maternal genetic score without or with adjustment for fetal genetic score (method 1 and 2) and maternal nontransmitted haplotype score (method 3). The results strongly suggested that the observed correlations between maternal height and birth length or birth weight were mainly due to fetal genetic effects but not due to nongenetic intrauterine effect (i.e., maternal height as an environmental factor that casually influences fetal growth). Multiple lines of evidence consistently supported this conclusion.

First, we observed significant association of fetal genetic score with birth length and birth weight, and after controlling for fetal genetic score, the associations between maternal genetic score with birth length and weight were no longer significant, which suggests substantial fetal genetic influence. This observation was in line with previous studies, which showed that the magnitudes of associations between paternal height with either birth length or birth weight were comparable to maternal height [20,21,64]. Genetic analyses of parent-offspring data [6,11] also suggested fetal genetics explain more variation in birth size.

Second, our haplotype-based genetic score analysis showed that both maternal transmitted genetic score and paternal transmitted genetic score were highly significantly associated with birth length and birth weight, but the nontransmitted score was not (particularly for birth weight), which again indicated that maternal height has relatively minimal effect on birth length or birth weight if not transmitted genetically. The nontransmitted score was marginally associated with birth length, which suggests some direct causal effect of maternal height on birth length, and the estimated causal effect size was similar to the observational effect (~0.05 cm change in birth length per 1 cm change in maternal height).

Thirdly, our single marker analysis indicated that those adult height-associated SNPs were excessively associated with birth length and birth weight in fetus, but the estimated effect sizes of these SNPs on birth length and birth weight were not well correlated with their effect on maternal height, which indicated direct but different functional effects of these SNPs on fetal growth rather than a common causal effect mediated through maternal height. The inference of the parental transmission in mother-child pairs also allows us to examine parent-of-origin effects of those height-associated SNPs on birth length and weight. Our results showed excessive differences between the maternal and paternal transmitted alleles. For birth weight, the effect sizes of the maternal transmitted alleles were generally larger than paternal transmitted ones.

We also performed the analysis using gestation-adjusted birthweight percentiles (converted to z-scores) as the outcome and obtained similar results (S8 Table). This evidence collectively suggests that birth weight and birth length are mainly defined by fetal genetics. Therefore, it is difficult to define abnormality of fetal growth in a general term (e.g., by deviation from population mean) without taking genetic variance into account. It should be noted that the samples of the current study were collected from high-resource northern European countries, with presumably good nutrition throughout childhood, adolescence, and pregnancy, enabling the mothers and their infants to grow to their full genetic potential. This fact might account for why we observed a predominant influence of genetic factors on birth size in this study. The conclusion may not be directly transferrable to low- or middle-income countries, where malnutrition may substantially constrain one’s growth and significantly change the association between maternal height and fetal growth [24,25].

In contrast with the findings in birth length and birth weight, we observed evidence that suggested gestational age at birth might be causally influenced by maternal height, in which case maternal height might operate as an important factor that shapes the intrauterine environment and influence the gestational age. Again, this conclusion was supported by multiple lines of evidence: (i) gestational age was (marginally) associated with maternal height genetic score but not with fetal height genetic score, and after adjustment for fetal genetics, the association between gestational age and maternal height genetic score were still significant; (ii) gestational age was associated with maternal nontransmitted haplotype genetic score, which indicated the effect of maternal height on gestational age was transmitted through phenotypic causal mechanism rather than genetic inheritance (i.e., transmission of alleles); (iii) the estimated causal effect of 0.4 d/cm of maternal height on gestational age was similar to the size from observational association; and (iv) single-marker analysis indicated that those adult height SNPs were excessively associated with gestational age, and the estimated effect sizes of these SNPs (in mothers) on gestational age, although relatively low, were correlated with their effect on maternal height, which suggested the effect of these SNPs is projected through a common intermediate (i.e., maternal height) rather than through SNP-specific pathways (biological pleiotropy).

We also performed analysis using preterm status as a dichotomous outcome, and the results (S9 Table) indicated that shorter maternal height is a risk factor for preterm birth (observational association *p* = 3.77 × 10^−6^, with an estimated 3% increase in risk if the mothers are 1 cm shorter). This result was consistent with a recent epidemiology study [18]. Genetic score analysis also showed significant association between preterm birth risk and the nontransmitted haplotype score (*p* = 0.0456), which suggests that maternal height causally influences preterm birth risk.

We recognize that the association between maternal non-transmitted haplotype score and gestational age was only modestly significant (meta *p* = 0.0424), and it was mainly driven by the DNBC cohort (*p* = 0.018) and was not significant in the other two cohorts. Also, gestational age in the DNBC cohort was estimated by combining information from self-reported data and gestational age reported in birth registry, while it was estimated by ultrasound in the other two cohorts. However, our meta-analysis did not reveal any sign of inconsistency—the confidence intervals of the estimates from different cohorts overlap and the *p*-value for the heterogeneity test was not significant. Nevertheless, the error-free validity of this association needs to be confirmed by further replication studies in independent cohorts.

### Using Parental Non-transmitted Haplotype Genetic Score in Causal Inference

Inference of parental transmission and analysis of parental-specific haplotype genetic scores is an important advantage of our current study. We are unaware of any published studies using this approach to disentangle the causal relationships between a parental exposure and outcomes in offspring. The distinction between the transmitted versus the nontransmitted maternal alleles can differentiate whether the causal mechanism is a phenotypic one (Fig 1A) or a genetic one (Fig 1C). Using nontransmitted haplotype genetic score can prevent the confounding due to genetic sharing between parents and offspring. Furthermore, the distinction between the maternal- and paternal-transmitted alleles can be used to study parent-of-origin effects, which are especially important in early-development phenotypes [65].

The maternal genotype-based genetic score (i.e., maternal height score) built on multiple associated SNPs, while capturing more variance in maternal height, is not a valid instrument (method 1) unless the genetic effect of those SNPs in offspring can be appropriately adjusted for. However, complete adjustment for the fetal genetic effect of multiple SNPs could be difficult to achieve. We recognized that the weighted height genetic score in infants might not correctly summarize the genetic effect of the height-associated SNPs on pregnancy outcomes, since the weights of the SNPs were based on their effect sizes on adult height. As indicated by our single SNP analyses, the estimated effect sizes of these SNPs on birth length and birth weight were poorly correlated with the reported effect sizes on adult height. Considering this fact, using maternal height genetic score as genetic instrument, even adjusted for offspring genotype by fetal genetic score (method 2), might be inappropriate and could lead to biased estimate in causal inference. In contrast, our approach that used the nontransmitted haplotype genetic score (method 3) did not suffer from this problem.

There are several caveats for our analytical approach: (1) high-density SNP data are required to construct long-range haplotypes in mothers and children when the allele transmission cannot be unambiguously determined from allelic states when both mother and child are heterozygote, and (2) in certain scenarios, the mutual independence between the transmitted and nontransmitted allele(s) cannot be assumed, e.g., in case of assortative mating dependent on the phenotype under study. The first limitation will require genotype data on additional SNPs around the SNPs used as genetic instrument, which is not a problem when genome-wide SNP data are available. The second limitation might introduce bias in causal inference, since the nontransmitted haplotype genetic score could correlate with the transmitted one. Intuitively, the extent of the bias due to assortative mating should be proportional to the correlation between haplotype genetic scores and the correlation between the (maternal and paternal) transmitted haplotype genetic scores and the pregnancy outcome, and therefore, it would be small because the correlation due to assortative mating is generally low.

Biological pleiotropy is always a potential issue that could undermine the validity of Mendelian randomization causal inference [38]. Complete exclusion of biological pleiotropy requires a strong assumption that the genetic instrument is associated with the outcome only through the intermediate phenotype but not through other biological mechanisms. Generally, it is not possible to prove this assumption [66], especially when a large number of genetic variants are used to construct the genetic instrument [43]. Some empirical evidences can help rule out major violations of the “no pleiotropy” assumption. For example, multiple genetic variants showing consistent results may support a common causal path through the intermediate phenotype that the variants are associated with [66]. In this study, the significant correlation between the estimated effect size of the height-associated SNPs on gestational age and the reported effect size on adult height strongly suggested consistent causal inference across multiple SNPs.

From a study design perspective, there are number of limitations of our study. We tried to infer a mechanistic causal relationship between maternal height and several pregnancy outcomes. For this type of study, a random sample from target population is usually preferred. The three included studies were all case/control studies, in which the samples were enriched for mother/child pairs with short gestational age (<37 wk). In addition, the case/control sampling also broke down the normal distributions of the traits, with the distribution of the gestational age broken down into two segments (one for controls and one for cases). Similarly, the distributions of birth length and birth weight have two modes. However, after adjustment for gestational age, birth weight and length followed normal distributions. Yet, there is no easy method to transform the two-segmented distribution of gestational age into normal. This problem might undermine the validity of our analysis of gestational age, although regression analysis is robust to the normality assumption [67]. To investigate whether it could be a significant problem, we tested the association between the nontransmitted haplotype score and gestational age using a nonparametric method, and the results showed similar significant association (meta *p* = 0.0053). In addition, we performed the same analysis only in term mother/child pairs (in which the gestational age followed a truncated distribution but did not significantly deviate from normal) and obtained similar results (meta *p*-v = 0.0270 for the association between gestational age and the maternal nontransmitted haplotype score).

Another potential problem is the differences in phenotype data collection and availability among the three studies. For example, gestational age at birth was estimated by last menstrual period in DNBC but by ultrasound in the MoBa and FIN studies, and birth length was unavailable in DNBC. In addition, the high missing rates in some of participant studies also prevented us from including some known important covariates like maternal BMI, smoking, and parity in the regression models. However, in the individual studies in which these variables were available, the analytical results from models with or without these covariates did not show significant differences. Therefore, we believe the major findings of our study are solid and robust to these limitations.

### Conclusion

In summary, this study defines the causal relationship for the strong association of maternal height with fetal growth measures (i.e., birth length and birth weight) and gestational age at birth. The observed association between maternal height and fetal growth parameters is mainly defined by fetal genetics, and many of the adult height-associated SNPs also influence fetal growth. In contrast, the association between maternal height and gestational age more likely reflects the maternal height phenotype, and the resulting fetal growth environment it shapes, as being deterministic. In addition, our approach that utilizes the genetic score derived from the parental nontransmitted haplotype as a genetic instrument is a novel extension to the Mendelian randomization methodology in casual inference between parental exposure and outcomes in offspring.

## Supporting Information

S1 Fig Q-Q plots showing excessive association between adult-height associated SNPs and birth length (A) and birth weight (B) in infants. (PDF)

S2 Fig Q-Q plot showing excessive association between adult-height associated SNPs and gestational length. (PDF)

S1 Table Descriptive statistics of maternal phenotypes. (PDF)

S2 Table Descriptive statistics of pregnancy outcomes. (PDF)

S3 Table Association between maternal genotype and haplotype genetic scores and maternal height. (PDF)

S4 Table Association between genotype height genetic scores and pregnancy outcomes. (PDF)

S5 Table Association between haplotype genetic scores and pregnancy outcomes. (PDF)

S6 Table Pairwise correlation (*p*-values) among genetic scores. (PDF)

S7 Table Results of instrumental variable analysis. (PDF)

S8 Table Statistical analyses using gestation-adjusted birth weight z-score as outcome. (PDF)

S9 Table Statistical analyses using dichotomous preterm birth as outcome. (PDF)

## Abbreviations:

ALSPAC: Avon Longitudinal Study of Parents and Children
BMI: body-mass index
dbGaP: Database of Genotypes and Phenotypes
DNBC: Danish National Birth Cohort
FIN: Finnish (Helsinki) Birth Cohort
GCTA: genome-wide complex trait analysis
GENEVA: Gene Environment Association Studies
GEI: Genes, Environment and Health Initiative
GWAS: genome-wide association study
IBD: identity by descent
LD: linkage disequilibrium
MoBa: Norwegian Mother and Child Cohort Study
PCA: principal component analysis
PPROM: preterm premature rupture of membranes
QC: quality control
Q-Q plot: quantile-quantile plot
SNP: single nucleotide polymorphism
