# Supplementary material for: Assessing the Causal Relationship of Maternal Height on Birth Size and Gestational Age at Birth: A Mendelian Randomization Analysis
Source: PLoS Med. 2015 Aug 18;12(8):e1001865. doi: 10.1371/journal.pmed.1001865 (PMC4540580; doi:10.1371/journal.pmed.1001865)
Supplement: S1 Fig — (PDF) [file pmed.1001865.s001.pdf]

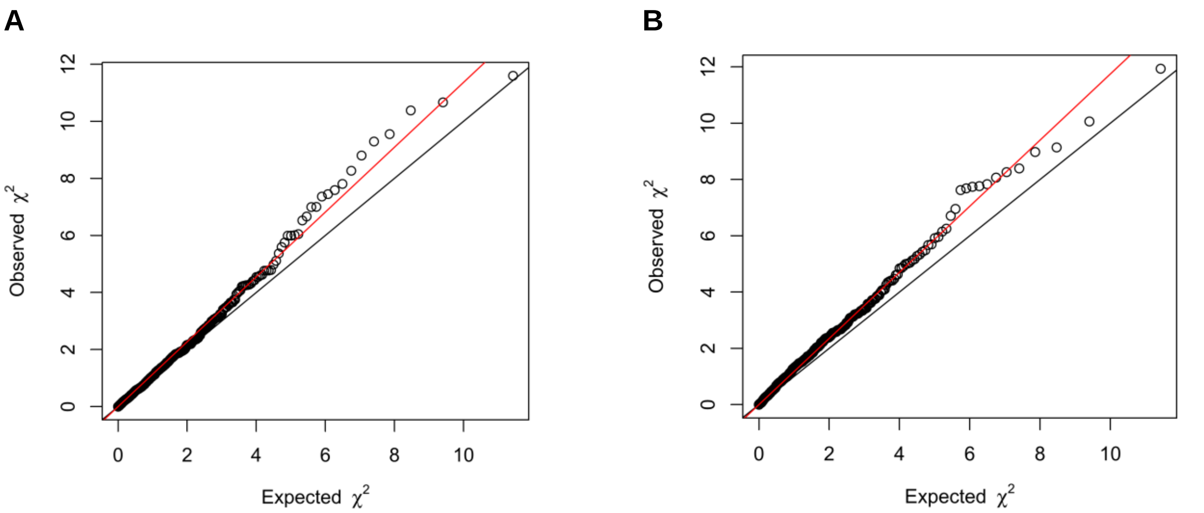

S1 Figure. Q-Q plots showing excessive association between adult-height associated SNPs and birth length (A) and birth weight (B) in infants.
