# Supplementary material for: Assessing the Causal Relationship of Maternal Height on Birth Size and Gestational Age at Birth: A Mendelian Randomization Analysis
Source: PLoS Med. 2015 Aug 18;12(8):e1001865. doi: 10.1371/journal.pmed.1001865 (PMC4540580; doi:10.1371/journal.pmed.1001865)
Supplement: S2 Fig — (PDF) [file pmed.1001865.s002.pdf]

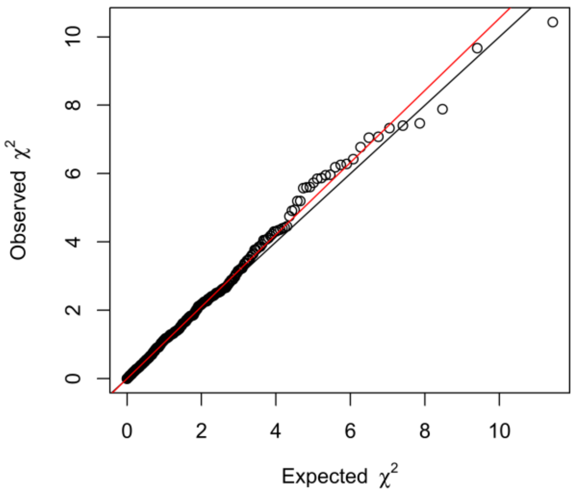

S2 Figure. Q-Q plot showing excessive association between adult-height associated SNPs and gestational age.
