# Supplementary material for: Assessing the Causal Relationship of Maternal Height on Birth Size and Gestational Age at Birth: A Mendelian Randomization Analysis
Source: PLoS Med. 2015 Aug 18;12(8):e1001865. doi: 10.1371/journal.pmed.1001865 (PMC4540580; doi:10.1371/journal.pmed.1001865)
Supplement: S1 Table — (PDF) [file pmed.1001865.s003.pdf]

S1 Table. Descriptive statistics of maternal phenotypes

FIN

Trait	all			normal			preterm		
	mean	sd	missing	mean	sd	missing	mean	sd	missing
age	31.29	4.389	0	31.43	4.202	0	30.98	4.781	0
height	166.5	5.869	0	166.7	5.814	0	166	5.972	0
weight	63.91	11.24	1	63.76	10.71	0	64.23	12.39	1
BMI	23.04	3.814	1	22.91	3.478	0	23.33	4.483	1

MoBa

trait	all			normal			preterm		
	mean	sd	missing	mean	sd	missing	mean	sd	missing
age	28.64	3.59	0	28.97	3.426	0	28.29	3.728	0
height	167.9	5.845	34	168.5	5.893	18	167.2	5.721	16
weight	67.64	12.1	46	68.11	11.79	26	67.15	12.41	20
BMI	24.02	4.197	51	24	4.021	28	24.03	4.379	23

DNBC

Trait	all			normal			preterm		
	mean	sd	missing	mean	sd	missing	mean	sd	missing
age	29.56	4.185	4	29.81	4.058	1	29.22	4.327	3
height	169	6.037	82	169.5	5.912	38	168.3	6.144	44
weight	66.98	12.69	108	67.5	12.06	51	66.28	13.47	57
BMI	23.44	4.216	109	23.5	4.014	52	23.37	4.479	57
