# Supplementary material for: Assessing the Causal Relationship of Maternal Height on Birth Size and Gestational Age at Birth: A Mendelian Randomization Analysis
Source: PLoS Med. 2015 Aug 18;12(8):e1001865. doi: 10.1371/journal.pmed.1001865 (PMC4540580; doi:10.1371/journal.pmed.1001865)
Supplement: S2 Table — (PDF) [file pmed.1001865.s004.pdf]

S2 Table. Descriptive statistics of pregnancy outcomes

FIN

Pregnancy outcomes	normal			preterm		
	mean	sd	missing	mean	sd	missing
Gestational days	282.1	6.48	0	238.5	12.38	0
Birth weight	3578	435	0	2408	487.2	0
Birth length	50.33	1.89	0	45.34	2.713	2
z-score	-0.195	0.8841	0	-0.1515	1.049	0

MoBa

Pregnancy outcomes	normal			preterm		
	mean	sd	missing	mean	sd	missing
Gestational days	280.3	3.644	0	248.2	11.34	0
Birth weight	3690	406.9	0	2743	463.4	0
Birth length	50.59	1.981	15	46.96	2.202	40

DNBC

Pregnancy outcomes	normal			preterm		
	mean	sd	missing	mean	sd	missing
Gestational days	282.3	3.846	0	239.8	15.44	0
Birth weight	3702	466.8	4	2457	652.9	9
