# Supplementary material for: Assessing the Causal Relationship of Maternal Height on Birth Size and Gestational Age at Birth: A Mendelian Randomization Analysis
Source: PLoS Med. 2015 Aug 18;12(8):e1001865. doi: 10.1371/journal.pmed.1001865 (PMC4540580; doi:10.1371/journal.pmed.1001865)
Supplement: S3 Table — (PDF) [file pmed.1001865.s005.pdf]

S3 Table. Association between maternal genotype and haplotype genetic scores and maternal height

cohort	Genotype				Transmitted				Un-transmitted			
	beta	se	p-val	r2	beta	se	p-val	r2	beta	se	p-val	r2
FIN	5.667	0.3973	**3.48E-41**	0.2085	6.097	0.5845	**6.22E-24**	0.1176	5.131	0.5717	**2.10E-18**	0.08872
MoBa	5.536	0.3469	**3.82E-51**	0.2071	6.029	0.5228	**6.14E-29**	0.1168	5.088	0.5004	**3.72E-23**	0.09236
DNBC	5.8	0.2888	**3.62E-80**	0.2015	5.975	0.4215	**4.87E-43**	0.1038	5.646	0.4102	**8.13E-41**	0.09819
meta	5.686	0.1938	**2.75E-189**		6.02	0.2861	**2.89E-98**		5.353	0.2774	**5.50E-83**	
p_het	0.8411				0.9856				0.6238			
