# Supplementary material for: Assessing the Causal Relationship of Maternal Height on Birth Size and Gestational Age at Birth: A Mendelian Randomization Analysis
Source: PLoS Med. 2015 Aug 18;12(8):e1001865. doi: 10.1371/journal.pmed.1001865 (PMC4540580; doi:10.1371/journal.pmed.1001865)
Supplement: S4 Table — (PDF) [file pmed.1001865.s006.pdf]

S4 Table. Association between genotype height genetic scores and pregnancy outcomes

FIN

Genotype score	Birth length			Birth weight			Gestational age		
	beta	se	p-val	beta	se	p-val	beta	se	p-val
Maternal	0.4444	0.1342	**0.0009706**	90.59	28.89	**0.001776**	0.1101	1.671	0.9475
Fetal	0.5617	0.1333	**2.79E-05**	93.91	28.79	**0.001153**	-0.4575	1.666	0.7837
Adjusted	0.2033	0.1574	0.1969	56.61	33.98	0.09611	0.4899	1.971	0.8037

MoBa

Genotype score	Birth length			Birth weight			Gestational age		
	beta	se	p-val	beta	se	p-val	beta	se	p-val
Maternal	0.3274	0.1252	**0.00908**	53.12	23.6	**0.0246**	0.9407	1.188	0.4285
Fetal	0.5104	0.1262	**5.68E-05**	76.46	24.04	**0.001515**	0.9272	1.213	0.4448
Adjusted	0.07299	0.1491	0.6245	17.69	28.04	0.5282	0.6349	1.415	0.6538

DNBC

Genotype score	Birth length			Birth weight			Gestational age		
	beta	se	p-val	beta	se	p-val	beta	se	p-val
Maternal	NA			86.67	22.73	**0.000142**	2.436	1.225	**0.04697**
Fetal				127.6	22.78	**2.51E-08**	0.1916	1.24	0.8772
Adjusted				26.07	26.83	0.3314	3.285	1.454	**0.02397**
