# Supplementary material for: Assessing the Causal Relationship of Maternal Height on Birth Size and Gestational Age at Birth: A Mendelian Randomization Analysis
Source: PLoS Med. 2015 Aug 18;12(8):e1001865. doi: 10.1371/journal.pmed.1001865 (PMC4540580; doi:10.1371/journal.pmed.1001865)
Supplement: S5 Table — (PDF) [file pmed.1001865.s007.pdf]

**S5 Table.** Association between haplotype genetic scores and pregnancy outcomes

***FIN***

| Haplotype score | Birth length | | | Birth weight | | | Gestational age | | |
|---|---|---|---|---|---|---|---|---|---|
| | beta | se | p-val | beta | se | p-val | beta | se | p-val |
| M1 (C1) | 0.5911 | 0.1969 | **0.002766** | 112.7 | 42.4 | **0.008025** | 0.2925 | 2.449 | 0.905 |
| M2 | 0.3215 | 0.1933 | 0.09677 | 74.4 | 41.57 | 0.0739 | 0.0004433 | 2.395 | 0.9999 |
| C2 | 0.5561 | 0.1895 | **0.003444** | 79.18 | 40.86 | 0.053 | -1.074 | 2.354 | 0.6485 |

***MoBa***

| Haplotype score | Birth length | | | Birth weight | | | Gestational age | | |
|---|---|---|---|---|---|---|---|---|---|
| | beta | se | p-val | beta | se | p-val | beta | se | p-val |
| M1 (C1) | 0.483 | 0.1868 | **0.009868** | 105.2 | 35.16 | **0.002846** | 0.516 | 1.774 | 0.7712 |
| M2 | 0.2254 | 0.1802 | 0.2111 | 12.23 | 34.04 | 0.7194 | 1.486 | 1.709 | 0.3848 |
| C2 | 0.5585 | 0.1767 | **0.001624** | 53.13 | 33.48 | 0.1129 | 1.347 | 1.683 | 0.4235 |

***DNBC***

| Haplotype score | Birth length | | | Birth weight | | | Gestational age | | |
|---|---|---|---|---|---|---|---|---|---|
| | beta | se | p-val | beta | se | p-val | beta | se | p-val |
| M1 (C1) | NA | | | 199.2 | 32.89 | **1.71E-09** | 0.8155 | 1.79 | 0.6487 |
| M2 | | | | -13.38 | 32.38 | 0.6795 | 4.106 | 1.738 | **0.01824** |
| C2 | | | | 64.12 | 32.57 | **0.04915** | -0.3333 | 1.758 | 0.8497 |
