# Supplementary material for: Assessing the Causal Relationship of Maternal Height on Birth Size and Gestational Age at Birth: A Mendelian Randomization Analysis
Source: PLoS Med. 2015 Aug 18;12(8):e1001865. doi: 10.1371/journal.pmed.1001865 (PMC4540580; doi:10.1371/journal.pmed.1001865)
Supplement: S6 Table — (PDF) [file pmed.1001865.s008.pdf]

S6 Table. Pairwise correlation (p-values) among genetic scores

FIN

score	score_m	score_m1	score_m2	score_c	score_c1	score_c2
score_m						
score_m1	0.7206 (0)					
score_m2	0.7329 (0)	0.0592 (0.09799)				
score_c	0.5335 (0)	0.7087 (0)	0.0744 (0.03737)			
score_c1	0.7206 (0)	1.0000 (0)	0.0592 (0.09799)	0.7087 (0)		
score_c2	0.0657 (0.06622)	0.0431 (0.2287)	0.0530 (0.1386)	0.7333 (0)	0.0431 (0.2287)	

MoBa

score	score_m	score_m1	score_m2	score_c	score_c1	score_c2
score_m						
score_m1	0.7214 (0)					
score_m2	0.7459 (0)	0.0769 (0.01413)				
score_c	0.5448 (0)	0.6945 (0)	0.1171 (0.0001808)			
score_c1	0.7214 (0)	1.0000 (0)	0.0769 (0.01413)	0.6945 (0)		
score_c2	0.0705 (0.02444)	0.0147 (0.6402)	0.0884 (0.004782)	0.7294 (0)	0.0147 (0.6402)	

DNBC

score	score_m	score_m1	score_m2	score_c	score_c1	score_c2
score_m						
score_m1	0.7090 (0)					
score_m2	0.7273 (0)	0.0318 (0.192)				
score_c	0.5379 (0)	0.7079 (0)	0.0733 (0.00262)			
score_c1	0.7090 (0)	1.0000 (0)	0.0318 (0.192)	0.7079 (0)		
score_c2	0.0664 (0.006406)	0.0211 (0.3861)	0.0732 (0.002642)	0.7209 (0)	0.0211 (0.3861)	
