# Supplementary material for: Assessing the Causal Relationship of Maternal Height on Birth Size and Gestational Age at Birth: A Mendelian Randomization Analysis
Source: PLoS Med. 2015 Aug 18;12(8):e1001865. doi: 10.1371/journal.pmed.1001865 (PMC4540580; doi:10.1371/journal.pmed.1001865)
Supplement: S7 Table — (PDF) [file pmed.1001865.s009.pdf]

S7 Table. Results of instrumental variable analysis

FIN

Method	Birth length			Birth weight			Gestational age		
	beta	se	p-val	beta	se	p-val	beta	se	p-val
Method 1	0.07828	0.0232	**0.0007387**	15.96	4.993	**0.001389**	0.0194	0.2935	0.9473
Method 2	0.04065	0.03092	0.1886	11.34	6.674	0.0894	0.09793	0.3921	0.8028
Method 3	0.05881	0.0345	0.08825	13.6	7.421	0.06679	8.11E-05	0.4369	0.9999
Method 4	0.05265	0.03712	0.156	12.87	8.005	0.108	0.006564	0.4717	0.9889

MoBa

Method	Birth length			Birth weight			Gestational age		
	beta	se	p-val	beta	se	p-val	beta	se	p-val
Method 1	0.06086	0.02226	**0.006267**	9.617	4.29	**0.02497**	0.1906	0.2162	0.378
Method 2	0.0198	0.02956	0.503	3.281	5.696	0.5646	0.1302	0.2873	0.6504
Method 3	0.04244	0.03225	0.1882	1.883	6.22	0.7621	0.2134	0.3105	0.492
Method 4	0.03174	0.03566	0.3735	-0.3423	6.919	0.9605	0.2043	0.3459	0.5548

DNBC

Method	Birth length			Birth weight			Gestational age		
	beta	se	p-val	beta	se	p-val	beta	se	p-val
Method 1	NA			14.98	4.011	**0.0001872**	0.5046	0.2139	**0.01833**
Method 2				4.359	5.075	0.3904	0.7476	0.269	**0.005449**
Method 3				-3.92	5.847	0.5026	0.864	0.302	**0.004224**
Method 4				-5.984	6.064	0.3237	0.909	0.3156	**0.003974**
