# Supplementary material for: Assessing the Causal Relationship of Maternal Height on Birth Size and Gestational Age at Birth: A Mendelian Randomization Analysis
Source: PLoS Med. 2015 Aug 18;12(8):e1001865. doi: 10.1371/journal.pmed.1001865 (PMC4540580; doi:10.1371/journal.pmed.1001865)
Supplement: S8 Table — (PDF) [file pmed.1001865.s010.pdf]

S8 Table. Statistical analyses using gestation adjusted birth weight z-score as outcome

A. Association between maternal height and birth weight z-score

Cohort	Preterm birth risk		
	beta	se	p-val
FIN	0.03026	0.006361	2.33E-06
MoBa	0.01747	0.005614	**1.91E-03**
DNBC	0.02573	0.004304	**2.77E-09**
meta	0.02437	0.003009	**5.49E-16**
p_het	0.2912		

B. Association between genotype height genetic scores and birth weight z-score

Genotype score	FIN			MoBa			DNBC			meta			
	beta	se	p-val	beta	se	p-val	beta	se	p-val	beta	se	p-val	p_het
Maternal	0.2243	0.07583	**0.00319**	0.1553	0.06581	**0.01848**	0.2005	0.05211	**0.000124**	0.1924	0.03597	**8.88E-08**	0.7715
Fetal	0.2409	0.07528	**0.001426**	0.2138	0.06706	**0.001477**	0.2984	0.05229	**1.36E-08**	0.2605	0.03616	**5.85E-13**	0.5832
Adjusted	0.1332	0.08927	0.1361	0.0589	0.07825	0.4518	0.05818	0.0615	0.3442	0.07541	0.04252	0.07611	0.7626

C. Association between haplotype genetic scores and birth weight z-score

Haplotype score	FIN			MoBa			DNBC			meta			
	beta	se	p-val	beta	se	p-val	beta	se	p-val	beta	se	p-val	p_het
M1 (C1)	0.2787	0.1113	**0.0125**	0.3034	0.09809	**0.002035**	0.4629	0.07546	**1.07E-09**	0.3756	0.05269	**1.01E-12**	0.2675
M2	0.184	0.1091	0.09213	0.03975	0.09499	0.6757	-0.03214	0.07422	0.665	0.03727	0.05155	0.4697	0.2614
C2	0.2141	0.1067	**0.0451**	0.1404	0.09345	0.1332	0.1526	0.07479	**0.04152**	0.1631	0.05122	**0.001451**	0.8575

D. Results of instrumental variable analysis*

Haplotype score	FIN			MoBa			DNBC			meta			
	beta	se	p-val	beta	se	p-val	beta	se	p-val	beta	se	p-val	p_het
Method 1	0.03958	0.01313	**0.002565**	0.02778	0.0119	**0.01956**	0.03405	0.009059	**0.000171**	0.03356	0.006318	**1.08E-07**	0.7989
Method 2	0.02661	0.01751	0.1286	0.01094	0.01584	0.4898	0.00945	0.01141	0.4075	0.0136	0.008184	0.09667	0.7003
Method 3	0.03368	0.01952	0.0844	0.006078	0.01722	0.7241	-0.00922	0.01316	0.4835	0.00473	0.009218	0.6079	0.1892
Method 4	0.03159	0.02109	0.1341	-7.2E-05	0.01919	0.997	-0.01398	0.01364	0.3053	-0.00042	0.009833	0.9662	0.1927
