# Supplementary material for: Assessing the Causal Relationship of Maternal Height on Birth Size and Gestational Age at Birth: A Mendelian Randomization Analysis
Source: PLoS Med. 2015 Aug 18;12(8):e1001865. doi: 10.1371/journal.pmed.1001865 (PMC4540580; doi:10.1371/journal.pmed.1001865)
Supplement: S9 Table — (PDF) [file pmed.1001865.s011.pdf]

S9 Table. Statistical analyses using dichotomous preterm birth as outcome

A. Association between maternal height and preterm birth

Cohort	Preterm birth risk		
	beta	se	p-val
FIN	-0.02555	0.01424	0.0728
MoBa	-0.03318	0.01178	**4.84E-03**
DNBC	-0.02932	0.009091	**0.001261**
meta	-0.0297	0.006423	**3.77E-06**
p_het	0.9167		

B. Association between genotype genetic scores and preterm birth

Genotype score	FIN			MoBa			DNBC			meta			
	beta	se	p-val	beta	se	p-val	beta	se	p-val	beta	se	p-val	p_het
Maternal	0.009925	0.1664	0.9524	-0.1059	0.1331	0.4262	-0.2039	0.1061	0.0547	-0.1308	0.07427	0.07815	0.542
Fetal	0.008501	0.1655	0.959	-0.1142	0.1361	0.4014	0.003803	0.1069	0.9716	-0.03104	0.07496	0.6788	0.7647
Adjusted	0.007507	0.1956	0.9694	-0.06419	0.1585	0.6855	-0.2904	0.1262	**0.02141**	-0.1599	0.08814	0.06957	0.3386

C. Association between haplotype genetic scores and preterm birth

Haplotype score	FIN			MoBa			DNBC			meta			
	beta	se	p-val	beta	se	p-val	beta	se	p-val	beta	se	p-val	p_het
M1 (C1)	0.1119	0.2442	0.6468	-0.1129	0.1988	0.5701	-0.09547	0.1544	0.5365	-0.05932	0.1091	0.5866	0.7338
M2	-0.09532	0.2376	0.6883	-0.1138	0.1916	0.5524	-0.3198	0.1508	**0.03395**	-0.212	0.106	**0.04559**	0.6021
C2	-0.09652	0.2337	0.6795	-0.1164	0.1887	0.5373	0.09564	0.1518	0.5286	-0.00987	0.1055	0.9255	0.6251

D. Results of instrumental variable analysis

Haplotype score	FIN			MoBa			DNBC			meta			
	beta	se	p-val	beta	se	p-val	beta	se	p-val	beta	se	p-val	p_het
Method 1	0.001748	0.0293	0.9524	-0.02275	0.02441	0.3514	-0.03912	0.01882	**0.03764**	-0.02587	0.01328	0.05147	0.4965
Method 2	0.001501	0.0391	0.9694	-0.01494	0.0324	0.6447	-0.06167	0.0237	**0.009253**	-0.03633	0.01718	**0.03447**	0.2842
Method 3	-0.01743	0.04344	0.6883	-0.01637	0.03505	0.6404	-0.06164	0.02644	**0.01973**	-0.03992	0.01898	**0.03551**	0.4979
Method 4	-0.01916	0.04693	0.6831	-0.01406	0.03906	0.7189	-0.06572	0.02769	**0.0176**	-0.04294	0.02035	**0.03488**	0.4769
